# Effect of tranexamic acid on mortality in patients with haemoptysis: a nationwide study

**DOI:** 10.1186/s13054-019-2620-5

**Published:** 2019-11-06

**Authors:** Takahiro Kinoshita, Hiroyuki Ohbe, Hiroki Matsui, Kiyohide Fushimi, Hiroshi Ogura, Hideo Yasunaga

**Affiliations:** 1 Department of Traumatology and Acute Critical Medicine, Osaka University Graduate School of Medicine, 2-15 Yamadaoka, Suita, 565-0871 Japan; 2 Department of Clinical Epidemiology and Health Economics, School of Public Health, The University of Tokyo, 7-3-1 Hongo, Bunkyo-ku, Tokyo, 113-0033 Japan; 3 Department of Health Policy and Informatics, Tokyo Medical and Dental University Graduate School of Medicine, 1-5-45 Yushima, Bunkyo-ku, Tokyo, 113-8519 Japan

**Keywords:** Haemoptysis, Tranexamic acid, Propensity score matching, Cost-effectiveness

## Abstract

**Background:**

Although tranexamic acid is widely used in patients with haemoptysis, whether it improves mortality has not been well investigated. The aim of this study was to evaluate the effect of tranexamic acid on in-hospital mortality among patients with haemoptysis.

**Methods:**

This was a retrospective study using data from the Japanese Diagnosis Procedure Combination inpatient database. We identified all cases of emergency admission due to haemoptysis from July 2010 to March 2017. Patients were divided into two groups: a control group, and a tranexamic acid group (those who received tranexamic acid on the day of admission). The primary outcome was in-hospital mortality, with secondary outcomes of hospital stay length and total healthcare cost. The data were evaluated using a propensity score matching analysis.

**Results:**

Among 28,539 included patients, 17,049 patients received tranexamic acid and 11,490 patients did not. Propensity score analysis generated 9933 matched pairs. Compared to the control group, patients in the tranexamic acid group had significantly lower in-hospital mortality (11.5% vs. 9.0%; risk difference, − 2.5%; 95% confidence interval (CI), − 3.5 to − 1.6%), shorter hospital stays (18 ± 24 days vs. 16 ± 18 days; risk difference, − 2.4 days; 95% CI, − 3.1 to − 1.8 days), and lower total healthcare costs ($7573 ± 10,085 vs. $6757 ± 9127; risk difference, $− 816; 95% CI, $− 1109 to − 523).

**Conclusions:**

Tranexamic acid may reduce in-hospital mortality among patients with haemoptysis requiring emergency admission.

## Background

Haemoptysis, defined as expectoration of the blood from the lower respiratory tract, is a potentially life-threatening condition with high morbidity and mortality [1, 2]. A recent French nationwide epidemiologic study that included more than 80,000 patients reported that 9.0% of patients who were hospitalised for haemoptysis required admission to the intensive care unit (ICU), and 8.7–10.1% of patients died during the admission. Hospital mortality was reported to be 5.9–7.1%, even after exclusion of deaths due to lung cancer, suggesting that haemorrhage control is essential [3].

Tranexamic acid is a synthetic derivative of the amino acid lysine, which blocks the interaction of plasminogen with the lysine residues of fibrin and exhibits an antifibrinolytic effect [4]. Many randomised controlled trials and meta-analyses have reported that tranexamic acid reduces blood loss or transfusion requirements during elective operations, including cardiac, orthopaedic, oral, gynaecological, and urological surgeries [5–7], and may even prevent death in patients with significant traumatic or postpartum haemorrhage [8–10]. However, the impact of tranexamic acid on the volume or duration of haemoptysis remains unclear, since relatively few studies have investigated the effects of tranexamic acid among haemoptysis patients, and the results of these studies were inconsistent [11–14]. Moreover, the potential mortality benefit of tranexamic acid in patients with haemoptysis has not yet been investigated.

We hypothesised that the administration of tranexamic acid would be effective for haemoptysis patients, as well as other acute haemorrhagic conditions, including trauma and postpartum haemorrhage. The aim of this study was to explore the effectiveness of tranexamic acid on mortality among patients with haemoptysis requiring emergency hospitalisation.

## Methods

The study was approved by the Institutional Review Board of the University of Tokyo. The board waived the requirement for informed consent because of the anonymous nature of the data, as no information on individual patients, hospitals, or treating physicians was obtained.

### Data source

We used the Japanese Diagnosis Procedure Combination inpatient database, which includes discharge abstracts and administrative claims data for more than 1200 acute care hospitals, and covers approximately 90% of all tertiary-care emergency hospitals in Japan. The database includes data on age, sex, body weight, body height, level of consciousness at admission, diagnoses, procedures, prescriptions, and discharge status. To optimise the accuracy of recorded diagnoses, the responsible physicians are obliged to record their diagnoses using standardised reference charts. The primary diagnoses are recorded using the International Classification of Diseases Tenth Revision (ICD-10) codes and text in the Japanese language. Furthermore, as the diagnostic records are linked to a payment system, the attending physicians are also required to report objective evidence for their diagnosis, for the purposes of treatment cost reimbursement [15]. A previous study of these diagnostic and procedural records has established the validity of the database [16], with diagnostic specificity exceeding 96% and sensitivity of 50–80%. The specificity and sensitivity of procedures both exceeded 90%.

### Study population

We identified all cases of emergency admission to ICUs or general wards due to haemoptysis in the database from July 2010 to March 2017.

All hospitalised patients who were diagnosed with haemoptysis (ICD-10 code R042) at admission to ICUs or general wards were included in the study. We did not include patients who developed haemoptysis after admission. We also excluded patients who were younger than 18 years of age, who died on the day of admission, who were discharged on the day of admission, or who were hospitalised electively. When patients were admitted with the code of haemoptysis more than once during the study period, we only used the data from the first admission.

### Group assignment

Patients who received intravenous tranexamic acid on the day of admission were defined as the tranexamic acid group, with the remaining patients defined as the control group.

### Covariates

Covariates included age, sex, smoking history (non-smoker, current/past smoker, missing data), body mass index at admission, Japan Coma Scale (JCS) at admission [17], Charlson comorbidity index [18], comorbid atrial fibrillation (I48), comorbid venous thromboembolism (I26, I80, I82, O22, O87, O88), comorbid chronic kidney disease (N18, T82.4, Z49.2, Z99.2), ambulance use, teaching hospital, ICU admission on the day of admission, aetiologies of haemoptysis (ICD-10 codes listed in Additional file 1), examinations on the day of admission (acid-fast bacilli culture, bronchoscopy, oesophagogastroduodenoscopy, and computed tomography), and treatments on the day of admission (therapeutic embolisation, dopamine use, adrenaline use, noradrenaline use, transfusion, oxygenation, mechanical ventilation, and renal replacement therapy).

Body mass index was categorised as < 18.5, 18.5–24.9, 25.0–29.9, ≥ 30.0 kg/m^2^, or missing data. JCS status was categorised as alert consciousness, dizziness, somnolence, or coma. JCS status was shown to be substantially correlated with the Glasgow Coma Scale [17]. The Charlson comorbidity index was scored according to the diagnoses for each patient and categorised as 0, 1, 2, 3, or≥ 4 [18]. Comorbidity with atrial fibrillation or venous thromboembolism was collected to predict possible use of anticoagulant agents prior to admission. Data on comorbid chronic kidney disease were collected because the treatment guidelines for tranexamic acid suggest that it should be administered cautiously in patients with renal impairment [19]. The aetiologies of haemoptysis were determined according to a previous epidemiologic study conducted using a French nationwide database [3].

### Outcomes

The primary outcome was in-hospital mortality. The secondary outcomes were length of hospital stay, discharged to home, and total healthcare cost of the admission. As the costs were recorded in yen, we converted them into US dollars (110 yen = $1 USD). For the assessment of the safety of tranexamic acid, we also collected data on post-admission complications from thromboembolism (I21–I22, I26, I63, D735, N280, K763, K550, I74, I80, I822, I823, and I829) and seizure (R568, R252, R568, G403, and G400) [20].

### Statistical analysis

A propensity score matching method was applied to compare the outcomes between the tranexamic acid group and the control group [21, 22]. A multivariable logistic regression model was employed to predict the patients’ propensity scores for tranexamic acid treatment. Predictor variables included age, sex, smoking history, body mass index, JCS, Charlson comorbidity index, comorbid atrial fibrillation, comorbid venous thromboembolism, comorbid chronic kidney disease, ambulance use, teaching hospital, ICU admission on the day of hospitalisation, aetiologies of haemoptysis, examinations on the day of admission, and treatments on the day of admission. One-to-one nearest neighbour matching without replacement was performed for the estimated propensity scores of the patients using a calliper width set at 20% of the standard deviation for the propensity scores [21–23]. To assess the performance of the matching, the baseline characteristics before and after propensity score matching were compared using absolute standardised differences, with an absolute standardised difference ≤ 10% considered to denote negligible imbalances between the tranexamic acid and control groups [24]. We performed a propensity score matching using the STATA module of PSMATCH2 provided by Leuven and Sianesi [25].

Crude outcomes were compared in the unmatched cohort using Student’s *t* tests for continuous variables and chi-square tests for categorical variables. In the matched cohort, we used a generalised estimating equation approach for comparisons of the primary and secondary outcomes, accompanied by cluster-robust standard errors with individual hospitals as clusters [26]. Risk differences and 95% confidence intervals (CIs) were calculated for the primary and secondary outcomes. These estimates were obtained by generalised estimating equation models using identity link functions, irrespective of the outcome types [27].

We conducted sensitivity analyses to confirm the robustness of the main result by applying different models, namely, propensity score adjustment analysis and stabilised inverse probability of treatment weighting analysis. First, for the overall cohort, we performed a multivariable regression model with generalised estimating equations accompanied by cluster-robust standard errors with hospitals used as the cluster variable. In this analysis, in-hospital mortality is defined as the dependent variable, and the intravenous tranexamic acid on the day of admission and the estimated propensity scores in the main analyses were used for covariates. Next, we applied a stabilised inverse probability of treatment weighting. The patient-specific stabilised inverse probability of treatment weighting was generated using the estimated propensity scores from the main analyses [28, 29].

As subgroup analyses, we evaluated treatment-by-covariate interactions to explore the heterogeneity of the treatment effects across the aetiologies: cryptogenic, tuberculosis, bronchopulmonary carcinoma, cystic fibrosis/bronchial dilatation, respiratory infection, and aspergillosis. Subgroup analyses were conducted in the propensity score-matched cohort only.

Continuous variables were presented as mean ± standard deviation, and categorical variables were described as numbers (%). All reported *p* values were two-sided, and *p* values < 0.05 were considered to be statistically significant. All analyses were performed using STATA/MP 15.0 software (StataCorp, College Station, TX, USA).

### Role of the funding source

The funding source had no role in the study design, data collection, data analysis, data interpretation, or writing of the report. The corresponding author had full access to all the study data and had final responsibility for the decision to submit for publication.

## Results

**Fig. 1:**
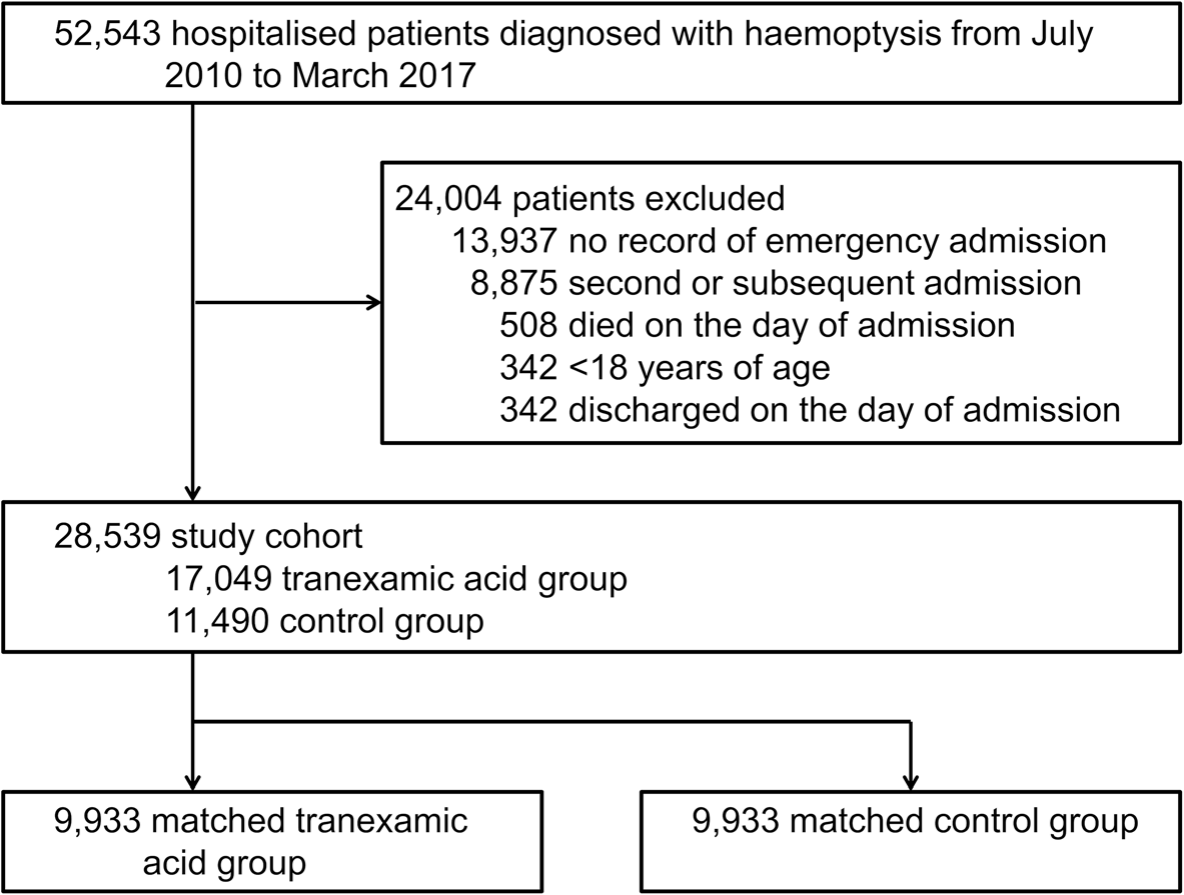
Flowchart of patient selection

We identified 52,543 hospitalised patients who were diagnosed with haemoptysis at admission from July 2010 to March 2017 (Fig. 1). A total of 28,539 patients met the inclusion criteria, including 17,049 patients who received tranexamic acid administration on the day of admission and 11,490 patients who did not. Among the former group, tranexamic acid was administered at a dose of 2 g or less in 16,325 (95.8%) patients. Among the control group, 2997 (26.1%) of patients received tranexamic acid treatment on the second day of admission or later.

A total of 9933 propensity score-matched pairs were generated from among the included patients. Table 1 shows the baseline characteristics of the unmatched and matched populations. In the unmatched cohort, there were several substantial differences (i.e. standardised differences of more than 10%) between the tranexamic acid group and the control group. Patients were more likely to receive tranexamic acid if they were younger, female, free of arterial fibrillation, transferred by ambulance, and treated in teaching hospitals. Patients with bronchopulmonary carcinoma or respiratory infection were less likely to receive tranexamic acid, while patients with cystic fibrosis/bronchial dilatation were more likely to receive tranexamic acid. Patients who received several examinations (including acid-fast bacilli culture, bronchoscopy, or computed tomography) and intensive treatments (including therapeutic embolisation and oxygenation) were more likely to receive tranexamic acid. After propensity score matching, all the baseline characteristics were well balanced, as indicated by the standardised differences.

**Table 1:** Patient characteristics at admission

Variables	Unmatched			Matched		
	Control (*n* = 11,490)	Tranexamic acid (*n* = 17,049)	Standardised difference (%)	Control (*n* = 9933)	Tranexamic acid (*n* = 9933)	Standardised difference (%)
Age, years	72 ± 14	71 ± 14	10	72 ± 14	72 ± 13	3
Male	7395 (64.4%)	9866 (57.9%)	13	6237 (62.8%)	6285 (63.3%)	1
Smoking history						
Non-smoker	5360 (46.6%)	8167 (47.9%)	3	4671 (47.0%)	4653 (46.8%)	0
Current/past smoker	4758 (41.4%)	7054 (41.4%)	0	4115 (41.4%)	4094 (41.2%)	0
Missing data	1372 (11.9%)	1828 (10.7%)	4	1147 (11.5%)	1186 (11.9%)	1
Body mass index, kg/m^2^						
< 18.50	3065 (26.7%)	4720 (27.7%)	2	2682 (27.0%)	2716 (27.3%)	1
18.50–24.99	5843 (50.9%)	8627 (50.6%)	1	5072 (51.1%)	5022 (50.6%)	1
25.00–29.99	1176 (10.2%)	1495 (8.8%)	5	962 (9.7%)	986 (9.9%)	1
≥ 30.00	221 (1.9%)	264 (1.5%)	3	176 (1.8%)	188 (1.9%)	1
Missing data	1185 (10.3%)	1943 (11.4%)	4	1041 (10.5%)	1021 (10.3%)	1
Japan Coma Scale on admission						
Alert	10,265 (89.3%)	15,582 (91.4%)	7	8948 (90.1%)	8892 (89.5%)	2
Dizziness	883 (7.7%)	1069 (6.3%)	6	705 (7.1%)	752 (7.6%)	2
Somnolence	155 (1.3%)	186 (1.1%)	2	126 (1.3%)	138 (1.4%)	1
Coma	187 (1.6%)	212 (1.2%)	3	154 (1.6%)	151 (1.5%)	0
Charlson comorbidity index						
0	4597 (40.0%)	7510 (44.0%)	8	4107 (41.3%)	3950 (39.8%)	3
1	3528 (30.7%)	5414 (31.8%)	2	3070 (30.9%)	3062 (30.8%)	0
2	1784 (15.5%)	2244 (13.2%)	7	1463 (14.7%)	1560 (15.7%)	3
3	690 (6.0%)	843 (4.9%)	5	552 (5.6%)	594 (6.0%)	2
≥ 4	891 (7.8%)	1038 (6.1%)	7	741 (7.5%)	767 (7.7%)	1
Comorbidity of atrial fibrillation	687 (6.0%)	665 (3.9%)	10	509 (5.1%)	530 (5.3%)	1
Comorbidity of venous thromboembolism	96 (0.8%)	102 (0.6%)	3	74 (0.7%)	88 (0.9%)	2
Comorbidity of chronic kidney disease	400 (3.5%)	333 (2.0%)	9	263 (2.6%)	284 (2.9%)	1
Ambulance transportation	3941 (34.3%)	6831 (40.1%)	12	3536 (35.6%)	3508 (35.3%)	1
Teaching hospital	8977 (78.1%)	14,822 (86.9%)	23	8239 (82.9%)	8073 (81.3%)	4
Intensive care unit admission	509 (4.4%)	789 (4.6%)	1	448 (4.5%)	461 (4.6%)	1
Aetiologies of haemoptysis						
Cryptogenic	3912 (34.0%)	5956 (34.9%)	2	3450 (34.7%)	3363 (33.9%)	2
Tuberculosis	543 (4.7%)	975 (5.7%)	5	490 (4.9%)	487 (4.9%)	0
Bronchopulmonary carcinoma	2181 (19.0%)	2511 (14.7%)	11	1771 (17.8%)	1851 (18.6%)	2
Cystic fibrosis/bronchial dilatation	1578 (13.7%)	3939 (23.1%)	24	1540 (15.5%)	1415 (14.2%)	4
Respiratory infection	4495 (39.1%)	5614 (32.9%)	13	3715 (37.4%)	3892 (39.2%)	4
Aspergillosis	479 (4.2%)	919 (5.4%)	6	453 (4.6%)	418 (4.2%)	2
Others	274 (2.4%)	283 (1.7%)	5	212 (2.1%)	235 (2.4%)	2
Examinations on the day of admission						
Acid-fast bacilli culture	4534 (39.5%)	9013 (52.9%)	27	4359 (43.9%)	4071 (41.0%)	6
Bronchoscopy	372 (3.2%)	1182 (6.9%)	17	368 (3.7%)	317 (3.2%)	3
Oesophagogastroduodenoscopy	368 (3.2%)	729 (4.3%)	6	353 (3.6%)	333 (3.4%)	1
Computed tomography	7008 (61.0%)	13,152 (77.1%)	36	6775 (68.2%)	6484 (65.3%)	6
Treatments on the day of admission						
Therapeutic embolisation	327 (2.8%)	893 (5.2%)	12	319 (3.2%)	294 (3.0%)	2
Dopamine use	101 (0.9%)	183 (1.1%)	2	95 (1.0%)	96 (1.0%)	0
Adrenaline use	123 (1.1%)	370 (2.2%)	9	123 (1.2%)	100 (1.0%)	2
Noradrenaline use	94 (0.8%)	112 (0.7%)	2	71 (0.7%)	79 (0.8%)	1
Transfusion	425 (3.7%)	637 (3.7%)	0	370 (3.7%)	380 (3.8%)	1
Oxygenation	3634 (31.6%)	6684 (39.2%)	16	3405 (34.3%)	3311 (33.3%)	2
Mechanical ventilation	414 (3.6%)	651 (3.8%)	1	367 (3.7%)	376 (3.8%)	1
Renal replacement therapy	83 (0.7%)	55 (0.3%)	6	44 (0.4%)	51 (0.5%)	1

Categorical variables are expressed as the number (%), and continuous variables are presented as the mean ± standard deviation. The total number of aetiologies does not add up to 100%, as more than one cause could be assigned to a single patient

Table 2 shows crude outcomes in the unmatched cohort. In-hospital mortality in the control group and the tranexamic acid group was 12.0% and 7.6%, respectively. Only a small proportion of patients were recorded as developing thromboembolism or seizure after admission in both groups.

**Table 2:** Comparison of crude outcomes between the two groups

	Control (*n* = 11,490)	Tranexamic acid (*n* = 17,049)	*p* value
Primary outcome			
In-hospital mortality	1379 (12.0%)	1290 (7.6%)	< 0.001
Secondary outcomes			
Length of hospital stay, days	19 ± 43	15 ± 17	< 0.001
Discharged to home	9398 (81.8%)	14,583 (85.5%)	< 0.001
Total health care cost for the admission, USD	7686 ± 11,009	6715 ± 8524	< 0.001
Thromboembolism	256 (2.2%)	313 (1.8%)	0.02
Seizure	9 (0.0%)	8 (0.0%)	0.29

Categorical variables are expressed as the number (%), and continuous variables are presented as the mean ± standard deviation. Student’s *t* tests were used for continuous variables, and chi-square tests were conducted for categorical variables

*USD* United States dollars

**Fig. 2:**
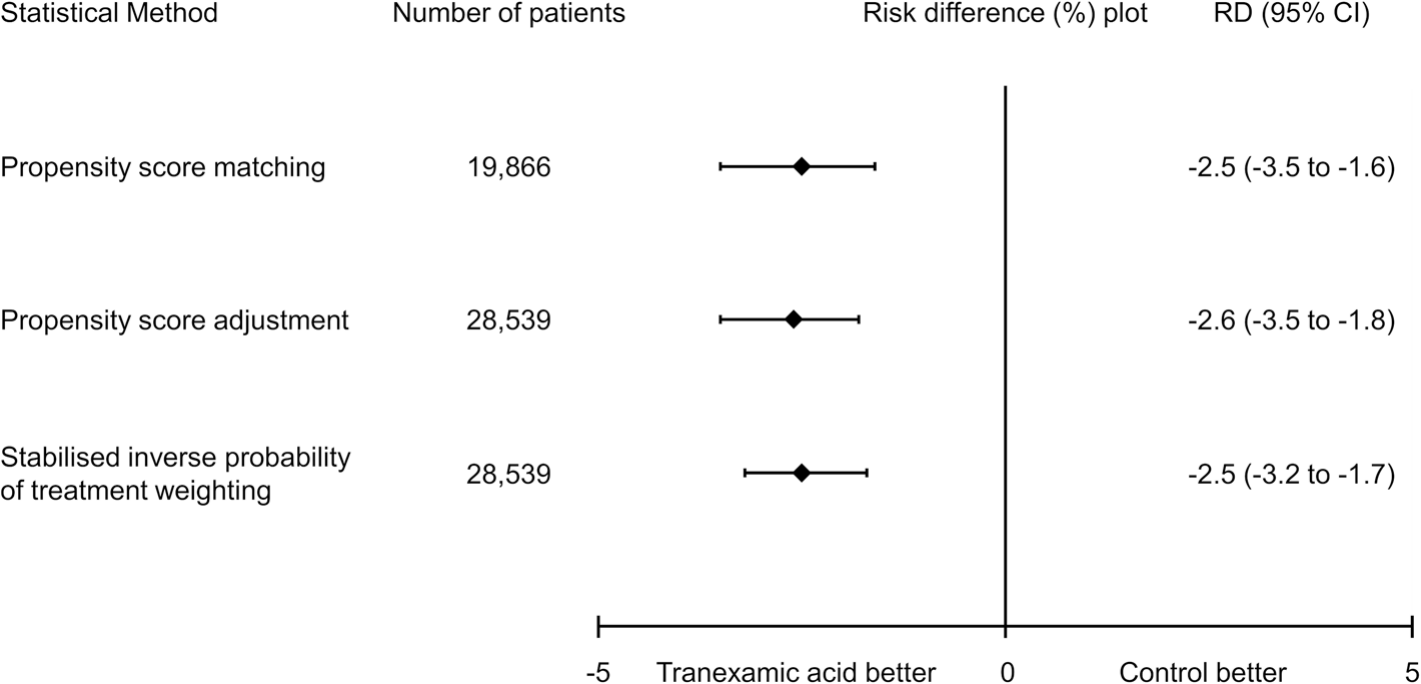
Association between tranexamic acid and in-hospital mortality applying three different propensity score models. Risk differences and 95% confidence intervals for in-hospital mortality by the treatment group using three models of propensity score analysis are shown. Propensity score matching shows the result of the propensity score matching analysis. Propensity score adjustment shows the result of the multivariable regression model using the estimated propensity score as a covariate. Stabilised inverse probability of treatment weighting shows the result of the stabilised inverse probability of treatment weighting analysis using estimated propensity scores. CI, confidence interval; RD, risk difference

After propensity score matching, patients in the tranexamic acid group had significantly lower in-hospital mortality than the control group (11.5% vs. 9.0%; risk difference, − 2.5%; 95% CI, − 3.5 to − 1.6%; *p* < 0.001) (Table 3). The estimated effect of tranexamic acid on in-hospital mortality according to the sensitivity analyses is summarised in Fig. 2. The significant association between the tranexamic acid group and reduced in-hospital mortality was consistent with the results found in the main analysis.

**Table 3:** Comparison of outcomes between the two groups in the matched cohort

	Matched			
	Control (*n* = 9933)	Tranexamic acid (*n* = 9933)	Risk difference (95% CI)	*p* value
Primary outcome				
In-hospital mortality	1141 (11.5%)	890 (9.0%)	− 2.5 (− 3.5 to − 1.6) %	< 0.001
Secondary outcomes				
Length of hospital stay, days	18 ± 24	16 ± 18	− 2.4 (− 3.1 to − 1.8)	< 0.001
Discharged to home	8124 (81.8%)	8410 (84.7%)	2.9 (1.7 to 4.1%)	< 0.001
Total health care cost for the admission, USD	7573 ± 10,085	6757 ± 9127	− 816 (− 1109 to − 523)	< 0.001
Thromboembolism	212 (2.1%)	232 (2.3%)	0.2 (− 0.2 to 0.6%)	0.34
Seizure	9 (0.0%)	5 (0.0%)		

Categorical variables are expressed as the number (%), and continuous variables are presented as the mean ± standard deviation. Risk difference and 95% confidence interval were obtained by generalised estimating equation models with identity link functions

*USD* United States dollars

**Fig. 3:**
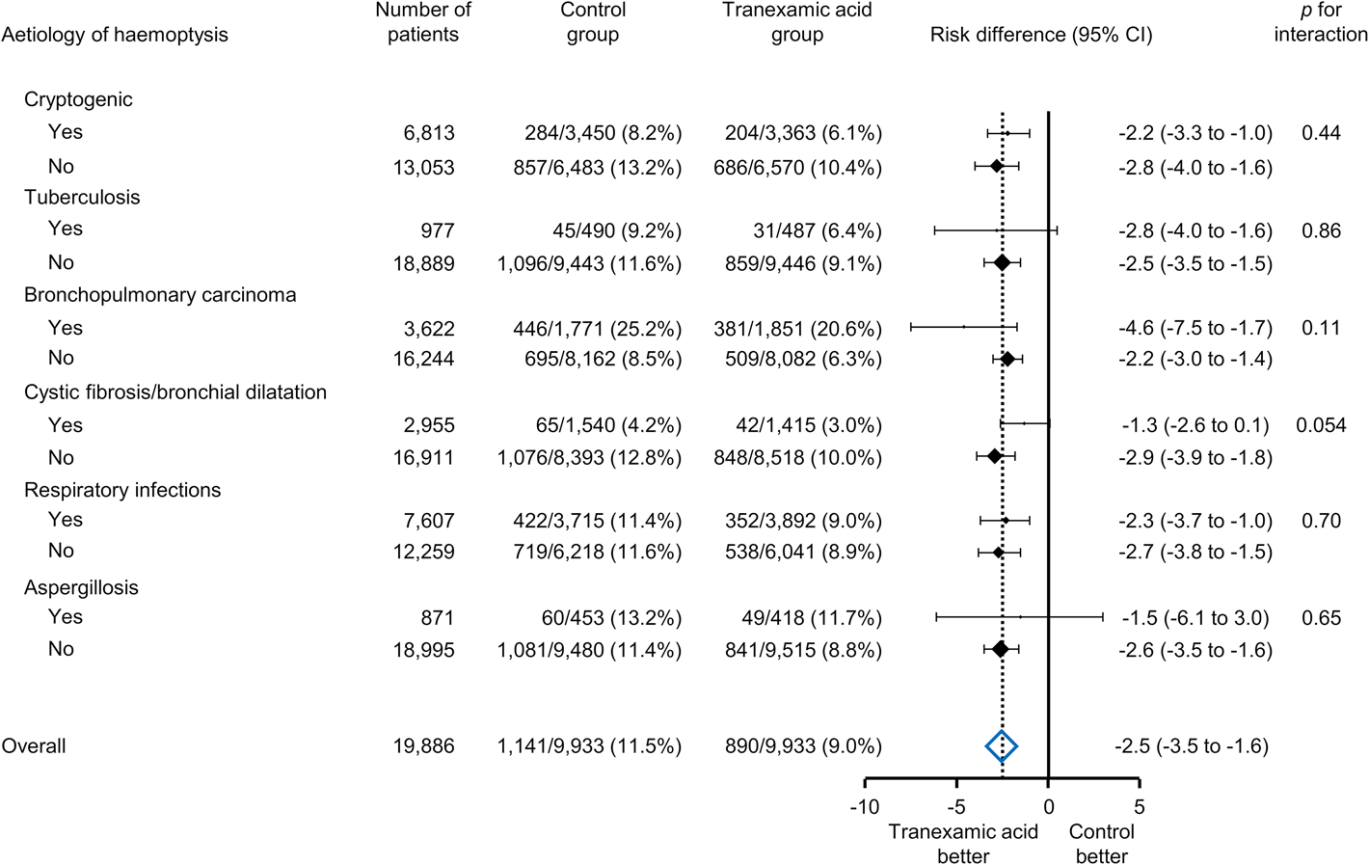
Subgroup analyses of in-hospital mortality. Risk differences for in-hospital mortality by the treatment group among all patients and within six subgroups of haemoptysis aetiology are shown. Risk differences and 95% confidence intervals were obtained by generalised estimating equation models with identity link functions. The size of the square represents the relative number within each subgroup, and the horizontal bars represent the 95% confidence interval. Tests for interactions were conducted for the subgroup analyses. CI, confidence interval

Hospital stays were significantly shorter in the tranexamic acid group compared to the control group (18 ± 24 days vs. 16 ± 18 days; risk difference, − 2.4 days; 95% CI, − 3.1 to − 1.8 days; *p* < 0.001). The proportion of patients discharged to home was significantly higher in the tranexamic acid group than in the control group (81.8% vs. 84.7%; risk difference, 2.9%; 95% CI, 1.7 to 4.1%; *p* < 0.001). Total healthcare costs for the admission were significantly lower in the tranexamic acid group than in the control group ($7573 ± 10,085 vs. $6757 ± 9127; risk difference, $− 816; 95% CI, $− 1109 to − 523; *p* < 0.001). There was no significant difference in the proportion of thromboembolism between the two groups (2.1% vs. 2.3%; risk difference, 0.2%; 95% CI, − 0.2 to 0.6%; *p* = 0.34). We could not conduct statistical testing for the complication of seizure between the two groups, due to the small number of events. With respect to in-hospital mortality, there were no significant interactions between the treatment group and any aetiology of haemoptysis (Fig. 3).

## Discussion

In this study, we investigated the effectiveness of tranexamic acid among haemoptysis patients requiring emergency admission. The major finding was that the administration of tranexamic acid was associated with lower in-hospital mortality, after adjustment for confounding using propensity score matching. To the best of our knowledge, this was the first study to report an effect of tranexamic acid on mortality in patients with haemoptysis.

Although the association between haemoptysis and haemostatic abnormalities has not been well investigated, haemoptysis potentially causes fibrinolysis, since 90% of haemorrhages originate from ruptured bronchial arteries, which contain high levels of tissue plasminogen activator (t-PA) in their endothelial cells [30]. Tranexamic acid is a well-known antifibrinolytic drug with established efficacy in prohibiting connections between fibrin and plasmin, which is activated by t-PA release from endothelial cells [4]. Based on this mechanism, tranexamic acid has been used in patients with haemoptysis to reduce the amount of expectorated blood, ostensibly by decreasing the fibrinolytic activity and thereby improving the clinical outcomes.

Previous randomised controlled trials that investigated the effect of tranexamic acid on haemoptysis were small; the numbers of enrolled patients ranged between 46 and 66 [11–14]. As such, one major strength of the current study was the larger sample size compared to previous studies. The absolute percentage of in-hospital mortality was 2.5% lower in the tranexamic acid group. Based on this figure, the number needed to treat in order to prevent one death from haemoptysis is 40. Furthermore, we believe that the number of propensity-matched patients in the present study (*n* = 19,866) is large enough to provide reasonably precise estimates of the effects of tranexamic acid.

Research in the field of severe trauma and postpartum haemorrhage strongly suggests that tranexamic acid reduces mortality when given within 3 h of onset, while it is no more effective when given later [9, 31]. Assuming this time-dependent effect modification in patients with haemoptysis, we conducted subgroup analyses investigating any difference in effect in six aetiology categories, including both acute and chronic onset diseases. However, the effect of tranexamic acid was not significantly different across acute diseases, such as respiratory infection and aspergillosis, and chronic conditions, such as tuberculosis, bronchopulmonary carcinoma, and cystic fibrosis/bronchial dilatation. Thus, we did not find sufficient evidence of a temporal effect of tranexamic acid in patients with haemoptysis. The association between the effects of tranexamic acid and timing of delivery should be investigated in future studies, controlling for the time from the onset of haemoptysis to the initiation of tranexamic acid treatment.

We also found that tranexamic acid was associated with shorter hospital stays and a higher probability of being discharged to home. These results were consistent with those in a recent randomised controlled trial, which reported that tranexamic acid inhalation was associated with significantly improved symptom resolution rates by 5days after admission, as well as shorter overall hospital stays [20]. Moreover, tranexamic acid was associated with lower total healthcare costs for the admission, indicating that the use of tranexamic acid in patients with haemoptysis may be dominant from the standpoint of cost-effectiveness. We speculated that reduced length of stay likely contributed to the decreased hospitalisation cost. Because the cost of administering tranexamic acid has been reported to be universally low ($17.5 in Tanzania to $48.0 in the UK) [32], we believe that our results in Japan could be generalisable to other countries.

Neither thromboembolism nor seizure was significantly increased by tranexamic acid. Previous randomised controlled trials for other diseases also showed that the number of thromboembolisms was not significantly different between the tranexamic acid group and the placebo group [5, 8, 9]. On the other hand, the risk of seizure associated with tranexamic acid was considered to be dose-dependent [5]. Administration of tranexamic acid at a dose of 100 mg/kg was significantly associated with seizure in one randomised controlled trial [5]; however, tranexamic acid did not increase seizure when it was used at a dose of 2 g or less [9]. The reason for the lack of association between tranexamic acid and seizure in the present study may be that most patients in the tranexamic acid group received ≤ 2 g of tranexamic acid.

The present study has several limitations. First, the retrospective and observational nature of the study design leaves it open to potential bias and confounding. However, we adjusted for measured confounders by propensity score matching using the generalised estimating equation approach. Although all measured variables were well-balanced, and hospital characteristics were adjusted for by the generalised estimating equation model, we could not adjust for unmeasured potential confounders such as vital signs, chest X-ray findings, and volume of haemoptysis, as these variables were not included in the database [33]. Second, it was also possible that anticoagulant-involved aetiology was not correctly recorded in the database, since its proportion was smaller than that of a previous study [3]. We attempted to adjust for the use of anticoagulant agents by including atrial fibrillation and venous thromboembolism as baseline variables. Third, the time from the symptom onset to the initiation of the tranexamic acid administration was not measured. The difference in the effects of tranexamic acid based on the timing of treatment should be investigated in future studies. Finally, the results of this study might have affected by patient crossover across the treatment groups; 26.1% of patients in the control group received tranexamic acid treatment on the second day of symptoms or later.

## Conclusions

This study, using a large nationwide database, suggested that administration of tranexamic acid was associated with reduced in-hospital mortality among patients with haemoptysis requiring emergency admission. This association should be confirmed in future randomised controlled trials.

## Supplementary information

**Additional file 1.** Aetiology of haemoptysis according to ICD-10 codes. The aetiology of haemoptysis was divided into the following categories: cryptogenic, tuberculosis, bronchopulmonary carcinoma, cystic fibrosis/bronchial dilatation, respiratory infection, aspergillosis, and “others”. Benign pulmonary bronchial tumour (D143), vasculitis (J991, M317, M310, and M301), pulmonary embolism (I260, I269, O880, O881, O883, and O888), thoracic trauma (S2580, S270, S272, S273, S275, S277, S278, S298, and S299), pulmonary oedema (I501, J81, I502, I081, I083, I342, I681, I050, and Q232), anticoagulant involvement (Y442 and D683), vascular malformation (Q252, Q254, Q257, Q258, I280, I719, and M352), bronchial endometriosis (N808), foreign body (T178, T174, and T175), and pulmonary haemosiderosis (E831) were defined as “others”, since only a small number of patients existed in each subgroup. Patients were classified into the cryptogenic category when none of the above listed diagnoses were recorded. ICD-10, International Classification of Diseases, Tenth Revision.

## Data Availability

The datasets used and analysed during the current study are available from the corresponding author on reasonable request.

## Abbreviations

ICU: Intensive care unit
ICD-10: International Classification of Diseases Tenth Revision
JCS: Japan Coma Scale
CIs: Confidence intervals
t-PA: Tissue plasminogen activator
