# Supplementary material for: Effect of tranexamic acid on mortality in patients with haemoptysis: a nationwide study
Source: Crit Care. 2019 Nov 6;23:347. doi: 10.1186/s13054-019-2620-5 (PMC6836388; doi:10.1186/s13054-019-2620-5)
Supplement: Supplementary file 1 — Additional file 1. Aetiology of haemoptysis according to ICD-10 codes. The aetiology of haemoptysis was divided into the following categories: cryptogenic, tuberculosis, bronchopulmonary carcinoma, cystic fibrosis/bronchial dilatation, respiratory infection, aspergillosis, and “others”. Benign pulmonary bronchial tumour (D143), vasculitis (J991, M317, M310, and M301), pulmonary embolism (I260, I269, O880, O881, O883, and O888), thoracic trauma (S2580, S270, S272, S273, S275, S277, S278, S298, and S299), pulmonary oedema (I501, J81, I502, I081, I083, I342, I681, I050, and Q232), anticoagulant involvement (Y442 and D683), vascular malformation (Q252, Q254, Q257, Q258, I280, I719, and M352), bronchial endometriosis (N808), foreign body (T178, T174, and T175), and pulmonary haemosiderosis (E831) were defined as “others”, since only a small number of patients existed in each subgroup. Patients were classified into the cryptogenic category when none of the above listed diagnoses were recorded. ICD-10, International Classification of Diseases, Tenth Revision. [file 13054_2019_2620_MOESM1_ESM.doc]

**Additional file 1: Aetiology of haemoptysis according to ICD-10 codes**

| Tuberculosis | |  |  | Others |  |  |
| --- | --- | --- | --- | --- | --- | --- |
|  | A150 | Pulmonary tuberculosis, confirmed on microscopic exam of expectoration, with or without culture | | | D143 | Benign tumor of bronchi and lungs |
|  | A151 | Pulmonary tuberculosis confirmed with culture only |  |  | J991 | Respiratory disorders in other diffuse connective tissue disorders |
|  | A152 | Pulmonary tuberculosis, with histological confirmation |  |  | M317 | Microscopic Micropolyangeitis |
|  | A155 | Tuberculosis of larynx, trachea and bronchi, with bacteriological and histological confirmation | | | M310 | Hypersensitivity angeitis |
|  | A156 | Tuberculous Pleurisy, with bacteriological and histological | |  | M301 | Polyarteritis with lung involvement [ChurgStrauss] |
|  | A157 | Tuberculosis primary infection of respiratory tract with bacteriological and histological confirmation | | | I260 | Pulmonary embolism, with acute cor pulmonale |
|  | A159 | Non–defined tuberculosis of the respiratory system, with bacteriological and histological confirmation | | | I269 | Pulmonary embolism (without acute cor pulmonale) |
|  | A162 | Pulmonary tuberculosis (with no mention of bacteriological and histological confirmation) | | | O880 | Obstetric air embolism |
|  | A164 | Tuberculosis of the larynx, trachea and bronchi (with no mention of bacteriological or histological confirmation) | | | O881 | Amniotic fluid embolism |
|  | A190 | Acute miliary tuberculosis, single location specified |  |  | O883 | Obstetrical Pyaemic and septic embolism |
|  | A191 | Acute miliary tuberculosis, multiple locations |  |  | O888 | Other obstetrical embolism |
|  | A192 | Unspecified acute miliary tuberculosis |  |  | S2580 | Injury of other thoracic blood vessels |
|  | A198 | Other miliary tuberculosis |  |  | S270 | Traumatic pneumothorax |
|  | A199 | Unspecified miliary tuberculosis |  |  | S272 | Traumatic Hemopneumothorax |
|  | B909 | Unspecified tuberculosis sequelae of respiratory tract |  |  | S273 | Other injuries of lungs |
|  |  |  |  |  | S275 | Injury of thoracic trachea |
| Bronchopulmonary carcinoma | | |  |  | S277 | Multiple injuries of intrathoracic organs |
|  | C341 | Malignant tumor of superior lobe, bronchi or lung |  |  | S278 | Injury of other specified intrathoracic organs |
|  | C342 | Malignant tumor of middle lobe, bronchi or lung |  |  | S298 | Other specified injuries of thorax |
|  | C343 | Malignant tumor of inferior lobe, bronchi or lung |  |  | S299 | Unspecified injury of thorax |
|  | C349 | Unspecified bronchi or lung malignant tumor |  |  | I501 | Left ventricular failure |
|  | C780 | Pulmonary metastasis of malignant tumor |  |  | J81 | Pulmonary edema |
|  | D381 | Neoplasm of uncertain behavior of trachea, bronchus and lung | | | I052 | Mitral stenosis (rheumatic) with insufficiency |
|  |  |  |  |  | I081 | Mitral and tricuspid involvement (rheumatic) |
| Cystic fibrosis/bronchial dilatation | | |  |  | I083 | Mitral, aortic and tricuspid valve involvement |
|  | J47 | Bronchiectasis |  |  | I342 | Non-rheumatic mitral valve stenosis |
|  | Q334 | Congenital bronchiectasis |  |  | I681 | Cerebral arteritis in infectious and parasitic diseases classified elsewhere |
|  | E840 | Cystic Fibrosis with pulmonary manifestations |  |  | I050 | Mitral stenosis |
|  |  |  |  |  | Q232 | Congenital mitral stenosis |
| Respiratory Infection | | |  |  | Y442 | Side effects of anticoagulant treatment during their therapeutic use |
|  | J40 | Bronchitis, (not specified as acute or chronic) |  |  | D683 | Hemorrhagic disorders due to circulating anticoagulants |
|  | J10 | influenza with pneumonia, other influenza virus identified | |  | Q252 | Aortic atresia |
|  | J11 | influenza with pneumonia, virus not identified |  |  | Q254 | Others congenital malformations of aorta |
|  | J12 | Adenoviral pneumonia |  |  | Q257 | Others congenital malformations of pulmonary artery |
|  | J13 | Pneumonia due to Streptococcus pneumoniae |  |  | Q258 | Others congenital malformations of great arteries |
|  | J14 | Pneumonia due to Haemophilus influenzae |  |  | I280 | Arteriovenous fistula of pulmonary vessels |
|  | J15 | Bacterial Pneumonia, not classified elsewhere |  |  | I719 | Aortic aneurysm of unspecified site (without mention of rupture) |
|  | J16 | Pneumonia due to other infectious organisms, not classified elsewhere | | | M352 | Behcet syndrome |
|  | J17 | Pneumonia in diseases classified elsewhere |  |  | N808 | Other endometriosis |
|  | J18 | Pneumonia due to unspecified organism |  |  | T178 | Foreign body in other and multiple parts of respiratory tract |
|  | J20 | Acute Bronchitis |  |  | T174 | Foreign body in trachea |
|  | A310 | Pulmonary Infection due to atypical mycobacteria |  |  | T175 | Foreign body in bronchus |
|  |  |  |  |  | E831 | Disorders of iron metabolism |
| Aspergillosis | |  |  |  |  |  |
|  | B440 | Invasive pulmonary Aspergillosis |  |  |  |  |
|  | B441 | Other pulmonary aspergillosis |  |  |  |  |
|  | B449 | Unspecified aspergillosis |  |  |  |  |

The aetiology of haemoptysis was divided into the following categories: cryptogenic, tuberculosis, bronchopulmonary carcinoma, cystic fibrosis/bronchial dilatation, respiratory infection, aspergillosis, and “others”. Benign pulmonary bronchial tumour (D143), vasculitis (J991, M317, M310, and M301), pulmonary embolism (I260, I269, O880, O881, O883, and O888), thoracic trauma (S2580, S270, S272, S273, S275, S277, S278, S298, and S299), pulmonary oedema (I501, J81, I502, I081, I083, I342, I681, I050, and Q232), anticoagulant involvement (Y442 and D683), vascular malformation (Q252, Q254, Q257, Q258, I280, I719, and M352), bronchial endometriosis (N808), foreign body (T178, T174, and T175), and pulmonary haemosiderosis (E831) were defined as “others”, since only a small number of patients existed in each subgroup. Patients were classified into the cryptogenic category when none of the above listed diagnoses were recorded.

ICD-10, International Classification of Diseases, Tenth Revision
